# Regulatory T Cells Are Protective in Systemic Inflammation Response Syndrome Induced by Zymosan in Mice

**DOI:** 10.1371/journal.pone.0064397

**Published:** 2013-05-10

**Authors:** Wenyuan Jia, Li Cao, Shuangwen Yang, Hailong Dong, Yun Zhang, Haidong Wei, Wei Jing, Lichao Hou, Chen Wang

**Affiliations:** 1 Department of Anesthesiology, Xijing Hospital, The Fourth Military Medical University, Xi'an, Shaanxi Province, China; 2 Department of Immunology, The Fourth Military Medical University, Xi'an, Shaanxi Province, China; 3 Department of Ultrasonography, Xijing Hospital, The Fourth Military Medical University, Xi'an, Shaanxi Province, China; University of Nebraska Medical Center, United States of America

## Abstract

Systemic inflammation response syndrome (SIRS) is a key and mainly detrimental process in the pathophysiology of multiple organ dysfunction syndrome. The balance of pro-inflammation and anti-inflammation controls the initiation and development of SIRS. However, the endogenous counterregulatory immune mechanisms that are involved in the development of SIRS are not well understood. CD4^+^CD25^+^Foxp3 (forkhead box P3)^+^ regulatory T lymphocytes (Treg cells) play a key role in the immunological balance of the body. Thus, our aim was to investigate the contribution of these key immunomodulators (Treg cells) to the immune dysfunction that is observed in zymosan-induced SIRS in mice. We first evaluated the level of Treg cells in the lung of mice 6 h, 1 d, 2 d, 3 d, 5 d, and 7 d after the injection of zymosan or normal saline by western blot, real-time PCR and flow cytometry. We found that the number of Treg cells and the levels of the Treg cell-related transcription factor (Foxp3) and cytokines (IL-10) in the zymosan-treated group significantly decreased on day 1 and day 2 and significantly increased on day 5 compared with the NS-treated group. In the next experiment, the mice were injected with 200 μg of anti-CD25 mAb (clone PC61) to deplete the Treg cells and then injected with zymosan 2 days later. The number of Treg cells decreased by more than 50% after the injection of the PC61 mAb. In addition, the expression of the anti-inflammatory cytokine IL-10 also decreased. Moreover, the depletion of the Treg cells profoundly increased the mice'mortality and the degree of lung tissue injury. In conclusion, Treg cells tend to play a protective role in pathogenesis of the zymosan-induced generalized inflammation, and IL-10 signaling is associated with their immunomodulatory effect.

## Introduction

Multiple organ dysfunction syndrome (MODS) is one of the major causes of death in the intensive care unit. This syndrome occurs after a variety of primary insults, including shock, trauma, burns, pancreatitis, and sepsis [1]. It is now widely recognized that systemic inflammation response syndrome (SIRS) is an important mechanism for MODS. During SIRS, innate and adaptive components are activated and induce a generalized inflammation that is characterized by a “cytokine storm” and other events that are characteristic of inflammation [2], [3]. Significant advancements in our understanding of SIRS pathophysiology and immune dysfunction have been made, but the endogenous counterregulatory immune mechanisms that are involved in the response against SIRS have not yet been elucidated. Regulatory T lymphocytes (Treg cells; CD4^+^CD25^+^Foxp3^+^cells) are known to suppress a wide range of immune responses through several different mechanisms, including the production of regulatory cytokines, the competition for essential cytokines, and contact-dependent mechanisms [4], [5]. Treg cells are of great importance in the defense against infection and the control of autoimmune diseases [6], [7], [8]. However, the role of Treg cells in SIRS is controversial. Several studies have suggested that the CD4^+^CD25^+^Treg cells from septic mice are better suppressors of the proliferation of T effector cells, but the Ab-mediated depletion of Treg cells does not alter the sepsis-induced mortality in the cecal ligation and puncture (CLP) model [9], [10]. Other studies have reported that the adoptive transfer of in vitro-stimulated Treg cells in both prevention and therapeutic modes significantly improved the survival of CLP mice [11], [12]. Additionally, the association of regulatory T cells with long-term immune dysfunction and severe sepsis has been reported [13]. There are several experimental animal models of SIRS/MODS, of which the zymosan-induced generalized inflammation (ZIGI) model and the cecal ligation and puncture (CLP) model are widely used. The ZIGI model induces pathological changes that mimic both the sequence and the intensity of human MODS of nonseptic origin [2], [14], [15], [16]. The CLP model attempts to mimic the pathophysiological changes that are typically observed in septic patients [17], [18]. Therefore, the pathological mechanisms of these changes are considerably different. Although the Treg-produced anti-inflammatory cytokine IL-10 is able to attenuate the development of MODS in the ZIGI model [19], [20], the role that Treg cells play during the zymosan-induced development of SIRS is unclear. We hypothesized that Treg cells may prevent the progression of SIRS. In the present study, the number and phenotype of the endogenous Treg cells in the ZIGI model were studied. In addition, the present study analyzed whether the Ab-mediated depletion of Treg cells affects the outcome and the development of SIRS.

## Materials and Methods

### Animals

Male BALB/c mice (20–25 g, 6 to 8 weeks-old, specific pathogen-free) were obtained from the Laboratory Animal Center of The Fourth Military Medical University. The animals were housed under standard conditions (free access to food and water, 12 h light/dark cycle at 20°C–25°C). All of the experimental protocols were approved by the Institutional Animal Care and Use Committee of The Fourth Military Medical University and were performed in accordance with the National Institutes of Health guidelines.

### Zymosan-induced generalized inflammation

Zymosan (ZYM; Z4250, Sigma, USA) was dissolved in normal saline (NS) to a final concentration of 25 mg/mL and was boiled at 100°C for 80 min. All of the suspensions were freshly made before use. Zymosan was intraperitoneally injected at a dose of 0.8 g/kg to induce the ZIGI model. The same volume of NS was administered intraperitoneally to the mice in the vehicle group.

### In vivo depletion of CD25^+^T cells

Unless otherwise stated, the mice were injected intraperitoneally with 200 μg of anti-CD25 mAb (clone PC61; Bio X Cell, USA), IgG1 (control isotype group; Bio X Cell, USA) or phosphate buffer solution (PBS, vehicle group) at day −2. Two days later (day 0), the mice were injected intraperitoneally with zymosan, as described above. The cell depletion was confirmed through the analysis of the CD4^+^CD25^+^cells and the CD4^+^Foxp3^+^cells in the spleen.

### Lung wet/dry weight ratio (W/D)

To quantify the magnitude of the pulmonary edema, we evaluated the lung W/D ratio. The harvested wet lung was weighed. It was then placed in an oven for 24 h at 80°C and re-weighed.

### Lung histology

After the lungs were removed from the mice, the tissue samples were fixed with 4% formalin for 48 h at 4°C embedded in paraffin, and sectioned into 4–6 μm thick slices. The slices were then stained with hematoxylin and eosin for histopathological observation.

### Flow cytometry

The spleens were harvested after the injection of zymosan or NS. The flow cytometry procedure was performed as described previously [21]. The total cell counts were obtained using a hemacytometer. All of the Abs were purchased from eBioscience (USA): anti-CD4 (RM4-5 conjugated to FITC), anti-CD25 (PC61 conjugated to APC), and anti-Foxp3 (FJK-16s conjugated to PE). For the Foxp3 staining, the extracellular staining and subsequent intracellular staining were performed according to the manufacturer's recommendations. The flow cytometry analysis was performed on a FACSAira cytometer (BD Bioscience, USA). The lymphocytes were identified by their forward scatter (FSC) and side scatter (SSC) characteristics, gated and further analyzed.

### Western blotting

The tissue samples were directly homogenized in Triton buffer (20 mM HEPES and 0.5% Triton X-100, pH 7.6). Aliquots of the protein extracts (50 mg) were separated on a 10% SDS-PAGE gel. Subsequently, the proteins were electrophoretically transferred onto a PVDF membrane (Bio-Rad, USA). The membranes were blocked with 5% non-fat milk in TBS-Tween 20 (TBST), incubated with the Foxp3 primary antibody (dilution 1∶200, 14-5773-82, eBioscience Inc., San Diego, CA, USA) overnight at 4 °C and then incubated with the horseradishperoxidase-conjugated secondary antibody (1∶3000, CWBio Co., Ltd., Beijing, China) in TBST for 2 h at room temperature. The blots were then developed using a chemiluminescent reagent (CWBio Co., Ltd., Beijing, China). To obtain equal protein loadings, the blots were restained with a GAPDH antibody (CWBio Co., Ltd., Beijing, China), which was used as a control.

### Quantitative real-time PCR

After collection, the lungs were snap-frozen in liquid nitrogen and stored at −80°C. The total RNA was extracted from the frozen tissue using the TRIzol reagent (TaKaRa, Japan) according to the manufacturer's instructions and stored at 4°C for future analysis. The cDNA was synthesized with the PrimeScript TM RT reagent Kit (DRR037S, TaKaRa, Japan). The quantitative real-time polymerase chain reaction (PCR) was performed using the SYBR® Premix Ex Taq™ (DRR081A, TaKaRa, Japan) in accordance with the manufacturer's protocol. Each set of experiments was performed three times. The 25 μl PCR reactions (12.5 μl SYBRgreen,1 μl PCR forward and 1 μl PCR reverse primers, and 2 μl cDNA) were maintained at 95°C for 30 s and then subjected to 40 cycles of 5 s at 95°C and 30 s at 60°C. The following primers were used (TaKaRa, Japan): mice Foxp3 F,5′-CAGCTCTGCTGGCGAAAGTG-3′and R, 5′-TCGTCTGAAGGCAGAGTCAGGA-3′; mice IL-6F, 5′-CCACTTCACAAGTCGGAGGCTTA-3′andR,5′-GCAAGTGCATCATCGTTGTTCATAC-3′;mice IL-10 F, 5′-GACCAGCTGGACAACATACTGCTAA-3′ and R,5′-GATAAGGCTTGGCAACCCAAGTAA-3′; mice TNF-αF,5′-GTTCTATGGCCCAGACCCTCAC-3′and R,5′ -GGCACCACTAGTTGGTTGTCTTTG-3′;miceGAPDH F,5′-TGTGTCCGTCGTGGATCTGA-3′ and R,5′-TTGCTGTTGAAGTCGCAGGAG-3′.

### Statistical analysis

All of the statistical analyses were performed using SPSS 16.0 (SPSS Inc., Chicago, IL, USA) and GraphPad Prism5. The histopathological scores are expressed as the median (range) and were analyzed using the Kruskal-Wallis H method followed by the Nemenyi test for multiple comparisons. All other values are expressed as the mean ± SEM and were analyzed by one-way ANOVA followed by the Newman-Keuls test for multiple comparisons. The survival curves were compared through the logrank test using the Prism software (GraphPad, San Diego, CA). Differences with a probability of *P*<0.05 were considered statistically significant.

## Results

### Changes in the lung and cytokines during the development of ZIGI

**Figure 1:**
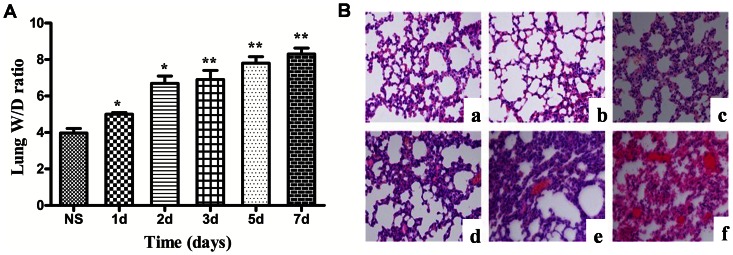
Lung W/D ratio (A) and histology (B). The lungs were stained with hematoxylin-eosin (original magnification: 40×). NS (a), 1d after the injection of zymosan (b), 2d after the injection of zymosan (c), 3d after the injection of zymosan (d), 5d after the injection of zymosan (e), and 7d after the injection of Zymosan (f). The values show the means ± SEM (n = 6 for each group). *P<0.05 compared with the NS-treated group; **P<0.01 compared with the NS-treated group.

**Figure 2:**
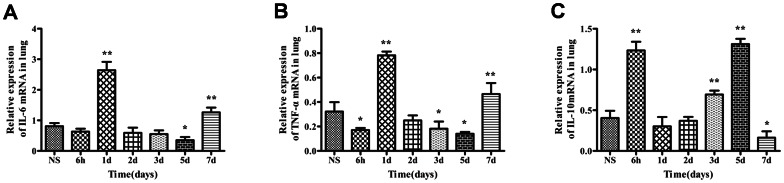
The IL-6, TNF-α and IL-10 mRNA expression levels in the lung tissues. Expression levels of IL-6 mRNA (A), TNF-α mRNA (B) and IL-10 mRNA in the lung tissues (C). The values show the means ± SEM (n = 6 for each group). *P<0.05 compared with the NS-treated group; **P<0.01 compared with the NS-treated group.

We tested the W/D ratio and the histopathology of the lung after the injection of zymosan or NS, as shown in Figure 1A. Compared with the mice that received NS, the mice the received zymosan exhibited edema of the lung, which progressed from day 1 to day 7. The histopathological analysis revealed that the zymosan-treated mice exhibited lung injury, which was characterized by alveolar wall thickening, the infiltration of neutrophils into the lung interstitium and the alveolar space and alveolar hemorrhage. As shown in Figure 1B and Table 1, the lung of the zymosan-treated group on day 1was not significantly different from the lung of the NS-treated group. However, from day 2 to day 7 after the injection of zymosan, the alveolar wall continued to thicken. In addition, worsening of the neutrophil infiltration and alveolar hemorrhage was observed during this time period, particularly in day7. The IL-6, TNF-α and IL-10 mRNA expression levels in the lung tissue were determined by real-time PCR. As shown in Figure 2A–C, the IL-6/GAPDH and TNF-α/GAPDH levels in the zymosan-treated mice increased significantly on day 1 (IL-6: 2.64±0.16; TNF-α: 0.78±0.03; *Ρ*<0.01) and day 7 (IL-6: 1.26±0.09; TNF-α: 0.46±0.05; *Ρ*<0.01) and decreased on day 5 (IL-6: 0.352±0.06; TNF-α: 0.14±0.01; *Ρ*<0.05) compared with the NS-treated group(IL-6: 0.81±0.10; TNF-α: 0.32±0.04). However, IL-10/GAPDH was found to be increased at 6 h (1.23±0.06; *Ρ*<0.01), day 3 (0.69±0.03; *Ρ*<0.01) and day 5 (1.31±0.04; *Ρ*<0.01) and decreased on day 7 (0.16±0.04; *Ρ*<0.05) compared with the NS-treated group (IL-10: 0.40±0.08).

**Table 1:** Lung histopathological scores.

Group						
Organ	NS	1d	2d	3d	5d	7d
Lung	1.0(1–1)	1.0(1–1)	1.5(1–2)*	2.0(1–3)*	3.0(2–4)**	3.5(2–4)**

The values show the median (range) (n = 6 for each group). *P<0.05 compared with the NS-treated group; **P<0.01 compared with the NS-treated group.

### Changes in the Treg cells during the development of ZIGI

**Figure 3:**
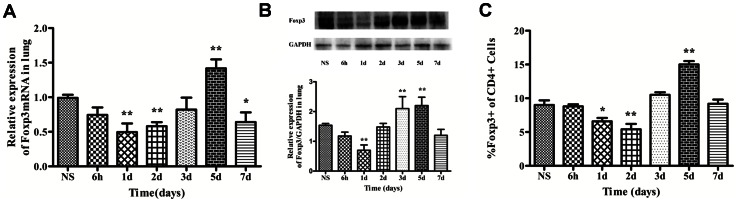
The Foxp3 mRNA expression level and the Foxp3 protein level in the lung tissues and flow cytometry analysis of the Treg cells in the spleen. Expression level of Foxp3 mRNA (A), Western blot of Foxp3 protein level (B) and flow cytometry of Treg cells (C). The values show the means ± SEM (n = 6 for each group). *P<0.05 compared with the NS-treated group; **P<0.01 compared with the NS-treated group.

To study whether Treg cells changed during the development of ZIGI, we tested the Treg cells after the injection of zymosan or NS by real-time PCR, western blotting and flow cytometry. The Foxp3 mRNA is the most specific transcription factor for Treg cells. We thus investigated the expression of Foxp3 mRNA. As shown in Figure 3A, the expression of Foxp3 mRNA in the zymosan-treated group on day 1 (0.49±0.07) was significantly decreased compared with the NS-treated group (0.99±0.04; *Ρ*<0.01). The Foxp3 mRNA expression after the zymosan injection then increased gradually on day 2, peaked at day 5 (1.41±0.07; *Ρ*<0.01), and began to decrease in day 7(0.64±0.08; *Ρ*<0.05). The Foxp3 protein expression level in the zymosan-treated group was decreased in day 1 (0.67±0.08; *Ρ*<0.01) and increased in day 3(2.13±0.21; *Ρ*<0.01) and day 5(2.18±0.12; *Ρ*<0.01) compared with the NS-treated group (1.51±0.04; Figure 3B). The numbers of CD4^+^Foxp3^+^cells within the CD4^+^population are shown in Figure 3C. As shown, the number of CD4^+^Foxp3^+^ cells in the zymosan-treated group was lowest on day 2 (5.40±0.80; *Ρ*<0.01) and then gradually increased until it peaked on day 5(15.00±0.50; *Ρ*<0.01) compared with the NS-treated group (9.00±0.70).

### The depletion of Treg cells was achieved in both normal and ZIGI mice

**Figure 4:**
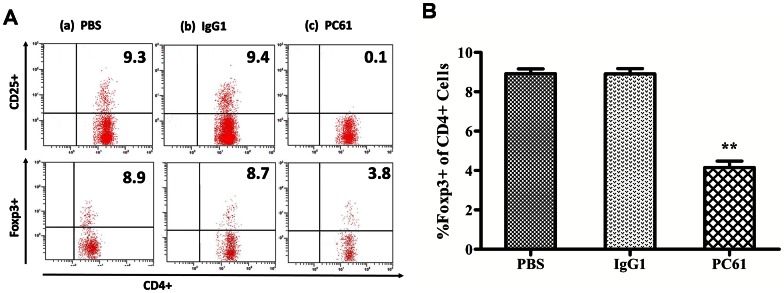
Effect of the injection of PC61 mAb on the Treg cells of normal mice. Mice (6 animals per group) were intraperitoneally injected with PBS (a), 200 μg of IgG1 mAb (b), or 200 μg of purified anti-CD25 mAb (PC61) (c). Two days after the injection, the spleen cells were collected. The numbers indicate the percentage of CD4^+^CD25^+^cells within the CD4^+^population and the percentage of Foxp3^+^cells within the CD4^+^population. The values show the means ± SEM. **P<0.01 compared with the PBS- and the IgG1-treated groups.

**Figure 5:**
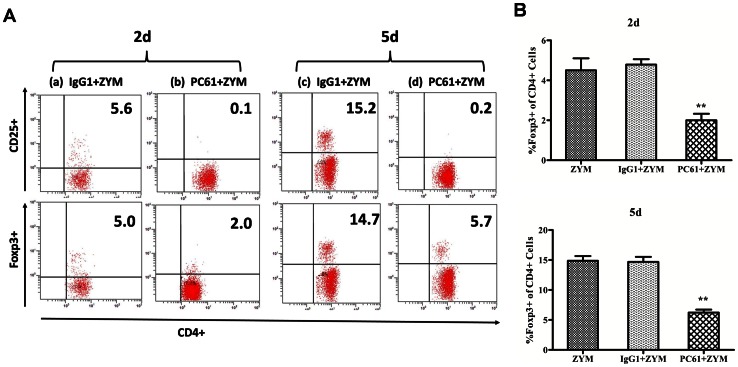
Effect of the injection of PC61 mAb on the Treg cells of ZIGI mice. Mice (6 animals per group) were injected intraperitoneally with 200 μg of IgG1 mAb (a, c) or 200 μg of purified anti-CD25 mAb (PC61) (b, d) on day −2. Zymosan was administered intraperitoneally on day 0. The spleen cells were collected on day 2 and day 5. The numbers indicate the percentage of CD4^+^CD25^+^cells within the CD4^+^population and the percentage of Foxp3^+^cells within the CD4^+^population. The values show the means ± SEM. **P<0.01 compared withthe ZYM and the IgG1+ZYM groups.

To analyze the role of Treg cells in mice, we performed depletion studies through the injection of PC61 mAb. Two days after the injection of this antibody, we analyzed the CD4^+^CD25^+^cells and the CD4^+^Foxp3^+^cells to determine if the depletion of these cells was achieved. As shown in Figure 4, the injection of 200 μg of the PC61 mAb lead to a significant depletion of the CD4^+^CD25^+^cells (0.02±0.20; *P*<0.01) and an approximately 50% depletion of the CD4^+^Foxp3^+^cells (4.15±0.32; *P*<0.01) in the spleen compared with the groups injected with PBS (CD4^+^CD25^+^cells: 9.20±0.34; CD4^+^Foxp3^+^ cells: 8.92±0.24) or the control isotype IgG1(CD4^+^CD25^+^cells: 9.30±0.30; CD4^+^Foxp3^+^ cells: 8.91±0.26). To study the role of Treg cells during SIRS, male mice, which had been previously injected with PC61 mAb to deplete the CD25^+^cells, were peritoneally injected with zymosan to induce the generalized inflammation model. The experimental groups in this analysis were the following: ZYM, IgG1+ZYM and PC61+ZYM. As shown in Figure 5, we found that the injection of the PC61 mAb depleted approximately 50% of the Foxp3^+^ cells that were present in the spleen on day 2 (2.02±0.32; *P*<0.01) and day 5(6.21±0.50; *P*<0.01) after the injection of zymosan compared with ZYM (day 2: 4.50±0.61; day 5: 14.87±0.79) and the IgG1+ZYM (day 2: 4.91±0.28; day 5: 14.70±0.82) groups.

### The depletion of Treg cells in mice worsens the lung injury, changes the expression of cytokines and decreases the survival rate

**Figure 6:**
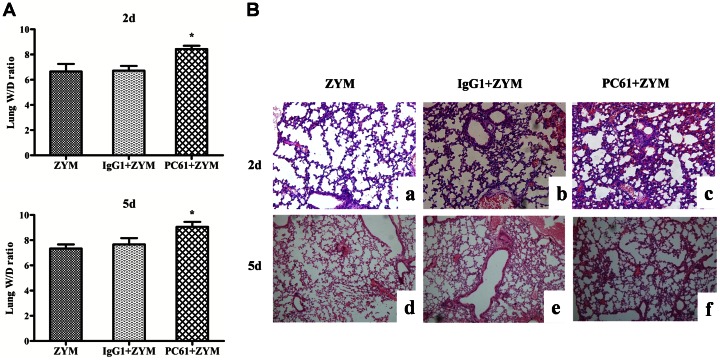
Lung W/D ratio and histology of ZIGI mice with depleted levels of Treg cells. Lung W/D ratio (A, n = 6 for each group). The lungs of ZIGI mice, which had depleted levels of Treg cells, were collected and stained with hematoxylin-eosin (B, original magnification: 10×). The values show the means ± SEM. *P<0.05compared with the ZYM and the IgG1+ZYM groups.

**Figure 7:**
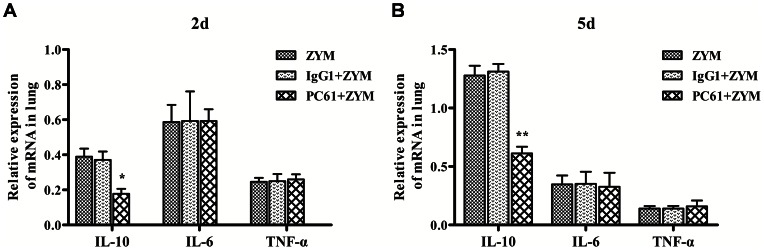
The IL-10, IL-6, and TNF-α mRNA expression levels in the lung tissues of ZIGI mice with depleted levels of Treg cells. Expression levels of IL-10 mRNA, IL-6 mRNA and TNF-α mRNA on day 2 (A) and day 5 (B) in the lung tissues of ZIGI mice with depleted levels of Treg cells. The values show the means ± SEM (n = 6 for each group). *P<0.05 compared with ZYM and IgG1+ZYM; **P<0.01 compared with ZYM and IgG1+ZYM.

**Figure 8:**
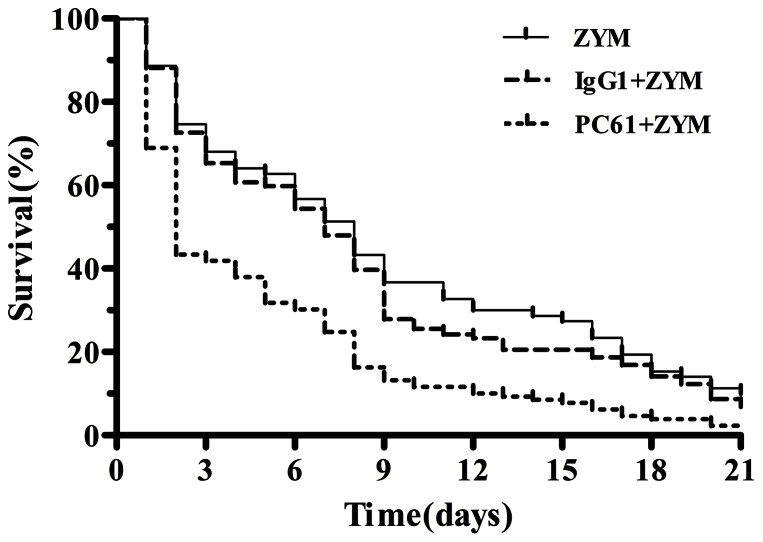
Survival of ZIGI mice with depleted levels of Treg cells. Survival of ZIGI mice that were administered the PC61 mAb. The following groups were analyzed: ZYM (n = 80), IgG1+ZYM (n = 80), and PC61+ZYM (n = 80). The survival curves were compared using the logrank test (*P*<0.05).

To determine the effect of the depletion of the Treg cells on the development of SIRS, the lung tissues were examined histologically, the cytokine expressions were detected by real-time PCR and the survival rates were assessed. The mice were pretreated with PC61 mAb 48 h prior to the induction of SIRS; their survival rate was assessed over 21 days, the histology of their lung tissues were observed on day 2 and day 5, and their cytokine mRNA levels were determined on day 2 and day 5. The depletion of the Treg cells aggravated the lung injury (Figure 6 and Table 2). The depletion of the Treg cells also decreased the IL-10 mRNA on day 2 (0.17±0.05; *P*<0.05) and day 5 (0.61±0.03; *P*<0.01) after the induction of ZIGI compared with the ZYM (day 2: 0.39±0.04; day 5: 1.27±0.05) and the IgG1+ZYM (day 2: 0.37±0.04; day 5: 1.31±0.04) groups, whereas no effect on the mRNA levels of IL-6 and TNF-α was observed (Figure 7). In addition, the mice that were not administered the PC61 mAb treatment exhibited a higher survival rate than the mice with depleted levels of Treg cells (*P*<0.05; Figure 8).

**Table 2:** Lung histopathological scores.

Group						
Organ	a	b	c	d	e	f
Lung	1.5(1–2)	1.5(1–2)	3.5(2–4)*	3.0(2–4)	3.0(2–4)	3.8(3–4)#

The values show the median (range) (n = 6 for each group). *P<0.05 compared with (a) and (b); #*P*<0.05 compared with (d) and (e).

## Discussion

Systemic inflammatory response syndrome (SIRS) is predominantly characterized by immunological disorder. SIRS is now believed to be the main mechanism for the development of MODS. The lung is the most vulnerable organ and is the first to fail during the development of MODS [16]. The intraperitoneal injection of zymosan initiates a triphasic inflammatory process that ultimately leads to the development of MODS [15]: phase I is an acute inflammatory response of sterile peritonitis, phase II is the recovery phase, and phase III is characterized by recurrent illness and progressive organ dysfunction. During the development of MODS, the symptoms of the mice are aggravated at first, then slightly relieved, and then aggravated again. However, the lung injury continually worsened throughout the process, which indicates that the inflammatory reaction that was induced by the immunological disorder continued to develop throughout the first 7 days.

The aim of this study was to observe the changes in the Treg cells during the development of SIRS and to determine whether this change was involved in the development of SIRS and the function of the Treg cells. The data obtained suggest that the development of SIRS changed the frequency of Treg cells. It was found that the number of Treg cells and the expression of the Treg cell-related transcription factor (Foxp3) in the zymosan-treated group was markedly decreased on day 1 and day 2 and significantly increased on day 5 compared with the NS-treated group. These data initially indicated the involvement of Treg cells in the zymosan-induced development of SIRS.

To explore the role of Treg cells in SIRS, the anti-CD25 mAb (PC61) was used to neutralize CD25. Two days after the administration of this treatment, a 50% reduction in the number of Treg cells was observed. In addition, the group that was administered the neutralizing antibody and zymosan exhibited a lower survival rate, aggravated lung injury, and a decreased induction of anti-inflammatory cytokines. Thus, we speculate that Treg cells may exert a protective role during the development of SIRS. In addition, this effect may be associated with the induction of IL-10. The day 2 and day5 time points were analyzed because the frequency of Treg cells significantly changed on these two days. The literature describes many protocols for the depletion of Treg cells; these include the use of a wide range of PC61 mAb concentrations (from 100 μg to 1 mg) in different models [8], [22], [23], [24]. To reduce the possibility of the elimination of activated T cells due to high antibody concentrations, we administered a PC61 mAb dose from the lower part of the reported range (200 μg) by intraperitoneal injection.

Several studies have suggested that the CD4^+^CD25^+^Treg cells from septic mice are better suppressors of the proliferation of T effector cells, but the Ab-mediated depletion of Treg cells does not affect the sepsis-induced mortality that is observed in the CLP model [9]. However, our present data differed from these results likely because of the different model that we used. The CLP model mimics the progression of sepsis, which is an infected inflammation, whereas the ZIGI model induces a sterile inflammation [14]. It was recently suggested that lymphocytes might not participate in the progression of sepsis in the CLP model. However, our data strongly suggest the involvement of Treg cells in the development of SIRS. This contradiction was caused by the depletion of different cell types of T lymphocytes. Markus Bosmann et al. hypothesized that T lymphocytes are not involved in the development of SIRS because the depletion of all lymphocytes did not influence the survival rate [25]. However, it is inappropriate to evaluate the mechanism of SIRS progression that results from the interaction of various types of T cells if all of the lymphocytes are depleted because some may relieve the injury and others may aggravate the development of SIRS.

Several recent studies have indicated the involvement of T lymphocytes in the development of SIRS. When innate immunity is induced, the infiltrating macrophages produce IL-23, which leads to the production of IL-17 by the infiltrating γδ T lymphocytes. IL- 17 then stimulates the macrophages and various other cells in the tissue to produce inflammatory factors [26]. During this period, the number of Treg cells decreases. Later in the infection, the proliferation of Treg cells is activated, which leads to immunosuppression. Although it has been verified that Treg cells may be involved in the development of SIRS through the exertion of a protective effect, the mechanism by which the multiple actions of potential factors interact to regulate the frequency of Treg cells (lowest on day 2 and highest on day 5) is unknown. Recently, Franziska Petermann et al. found that γδ T lymphocytes may suppress the proliferation of Treg cells and that this suppression is mediated by the release of heat-sensitive factors [27]. Our unpublished results show that the level of IL-17 peaked when the number of Treg cells was at its lowest level during the development of SIRS. It can be speculated that Treg cells may be regulated through γδ T lymphocytes. The present study enriched our knowledge on understanding of the function of T lymphocytes in the development of SIRS. A better understanding of the immunomodulatory effect of endogenous Treg cells may lead to the development of a novel therapeutic approach that can be used to decrease the mortality associated with SIRS/MODS.
